# Shotgun Metagenomics Versus 16S rDNA Sanger Sequencing for the Diagnosis of Blood Culture-Negative Endocarditis: A Draw?

**DOI:** 10.1093/ofid/ofag720

**Published:** 2026-11-02

**Authors:** Safa Ben Brahim, Guilhem Royer, Hervé Jacquier, Raphaël Lepeule, François Goehringer, Justine Cheval, Christophe Rodriguez, Corentine Alauzet, Paul-Louis Woerther

**Affiliations:** CHRU-Nancy, Laboratoire de Microbiologie, Université de Lorraine, Nancy, France; Stress Immunity Pathogens, Université de Lorraine, SIMPA, Nancy, France; Unité de Bactériologie, Département de Prévention, Diagnostic et Traitement des Infections, Hôpitaux Universitaires Henri Mondor, AP-HP, Créteil, France; Unité de Bactériologie, Département de Prévention, Diagnostic et Traitement des Infections, Hôpitaux Universitaires Henri Mondor, AP-HP, Créteil, France; INSERM U955, Team «Viruses, Cancer, Hepatology», Institut Mondor de Recherche Biomédicale, Université Paris Est Créteil, Créteil, France; Unité de Bactériologie, Département de Prévention, Diagnostic et Traitement des Infections, Hôpitaux Universitaires Henri Mondor, AP-HP, Créteil, France; INSERM U955, Team «Viruses, Cancer, Hepatology», Institut Mondor de Recherche Biomédicale, Université Paris Est Créteil, Créteil, France; INSERM U955, Team «Viruses, Cancer, Hepatology», Institut Mondor de Recherche Biomédicale, Université Paris Est Créteil, Créteil, France; Unité Transversale de Traitement des Infections, Département de Prévention, Diagnostic et Traitement des Infections, Hôpitaux Universitaires Henri Mondor, AP-HP, Créteil, France; Service de Maladies Infectieuses et Tropicales, CHRU Nancy, Nancy, France; Plateforme de Génomique GenoBioMICS, Hôpitaux Universitaires Henri Mondor, APHP, Créteil, France; INSERM U955, Team «Viruses, Cancer, Hepatology», Institut Mondor de Recherche Biomédicale, Université Paris Est Créteil, Créteil, France; Plateforme de Génomique GenoBioMICS, Hôpitaux Universitaires Henri Mondor, APHP, Créteil, France; CHRU-Nancy, Laboratoire de Microbiologie, Université de Lorraine, Nancy, France; Stress Immunity Pathogens, Université de Lorraine, SIMPA, Nancy, France; Unité de Bactériologie, Département de Prévention, Diagnostic et Traitement des Infections, Hôpitaux Universitaires Henri Mondor, AP-HP, Créteil, France; INSERM U955, Team «Viruses, Cancer, Hepatology», Institut Mondor de Recherche Biomédicale, Université Paris Est Créteil, Créteil, France

**Keywords:** infective endocarditis, 16S PCR, Sanger sequencing, shotgun metagenomics, molecular diagnostic

## Abstract

Although valuable for diagnosing blood culture-negative infective endocarditis, 16S rDNA sequencing and metagenomics on valve/vegetation have never been compared using identical DNA extracts. We compared methods on surgical samples from definite, possible, and rejected infective endocarditis involving culture-negative native valves. Positivity rates were similar, with taxonomic resolution being higher for metagenomics.

## INTRODUCTION

Infective endocarditis (IE) is a rare but severe pathology requiring rapid and reliable microbiological diagnosis. In blood culture-negative infective endocarditis (BCNIE), additional investigations such as serology or analysis of valve or vegetation samples may be necessary. However, prior antibiotic exposure, as well as IE caused by intracellular (eg *Bartonella* spp.) or fastidious bacteria (eg HACEK group bacilli), often results in negative cultures, making molecular approaches particularly valuable. Polymerase chain reaction (PCR) targeting the pan-bacterial 16S rDNA gene (16S PCR) remains the most widely used molecular approach in medical bacteriology laboratories. Following amplification, bacterial identification is usually achieved by Sanger sequencing. Next-generation sequencing (NGS) can also be used to sequence 16S rDNA, with greater specificity and the possibility of detecting mixed bacterial populations [1].

More recently, shotgun metagenomics (SMg) has emerged as a molecular tool to explore deep-seated infections and is now part of the 2023 Duke-ISCVID criteria for IE [2]. This pan-microbial approach can be very useful in serious infections. It can increase diagnostic sensitivity, improve species-level identification, provide complementary genomic information, and detect polymicrobial infections [3].

Several studies compared both methods with conventional bacterial approaches in the diagnosis of IE [1, 4], but direct comparison of SMg and 16S PCR-based approaches remains scarce. Some studies used both methods but included heterogeneous specimen types and did not consider IE classification [5–7]. In this context, we compared 16S PCR coupled with Sanger sequencing (16S PCR/SS) and SMg for the detection and identification of microorganisms on surgical samples from patients with native valve BCNIE in 2 French hospitals.

## MATERIALS AND METHODS

### Study Population and Samples

The cohort analyzed in this study was drawn from a French multicenter cohort, the French National Observatory on Infective Endocarditis (ClinicalTrials.gov ID: NCT03272724), which gathers all the data required (from hospital admission to discharge) to apply the 2015 ESC criteria [8]. Patients were informed of the study, and individual consents have been waived, in accordance with French legislation. Patients with a suspected native valve IE hospitalized in two participating centers, Nancy University Hospital and Henri Mondor University Hospital in Créteil, France, were prospectively enrolled from January 2022 to December 2023. All participants were ≥18 years of age and were covered by the French National Health Insurance System. Inclusion criteria were negative blood cultures and the availability of a culture-negative cardiac sample (valve and/or vegetation). Patients with positive blood and/or surgical sample cultures were excluded. IE were classified as definite, possible, or rejected, based on major and minor criteria as defined by the 2015 ESC guidelines [8].

The surgical samples were sent to the laboratories within 2 hours at room temperature. As part of routine bacteriological processing, samples were homogenized using an Ultra Turrax Tube Drive (IKA^®^) and subsequently frozen at −80 °C until DNA/RNA extraction and molecular biology analysis.

### Sample Treatment

#### Nucleic Acid Extraction

DNA and RNA were extracted using the QIAsymphony DSP Virus/Pathogen Kit on the QIAsymphony platform (Qiagen) following sample pretreatment with proteinase K and mechanical disruption by bead beating (glass beads; TissueLyser II), as previously described [9].

#### Amplification of the 16S rDNA Gene and Sanger Sequencing of the PCR Products

Amplification and Sanger sequencing of the 16S rDNA gene (16S PCR/SS) were performed at Nancy University Hospital's microbial genomics platform, as previously described [10]. Briefly, complete (≃1500 bp) and partial (≃500 bp) 16S rDNA were amplified using the primer pairs P8 (5′-AGTTTGATCCTGGCTCAG-3′)/P1525 (5′-AAGGAGGTGATCCAGCCGCA-3′) and P536 (5′-CAGCAGCCGCGGTAATAC-3′)/P1050 (5′-CAGGAGCTGACGACA-3′), respectively. When an amplicon was obtained using both primer pairs, the first 500 bp was sequenced using primers P8 and P519 (5′-GTATTACCGCGGCTGCTGGCAC-3′). When an amplicon was only obtained by using the primer pair P536/P1050, the latter was used for sequencing. For very low-intensity amplicons, a preliminary step of nested PCR was used. Sanger sequencing was performed on an automated sequencer, ABI PRISM 3100 (Applied Biosystems). The sequences were compared with the NCBI “core_nt” database using BLASTn. Interpretation and taxonomic affiliation were done according to the Clinical and Laboratory Standards Institute (CLSI) MM18 guideline [11], using a minimum sequence identity threshold of 99.0% for species-level identification and between 97.0% and 98.9% for genus-level identification.

#### Shotgun Metagenomics

SMg was performed on the Genobiomics platform of Henri Mondor University Hospital (Créteil, France), following previously described protocols and ISO 15189-certified diagnostic procedures [9]. This approach enables untargeted sequencing of both DNA and RNA from clinical samples. No enrichment, depletion, or amplification steps were performed. PCR amplification was only performed as part of the DNA and RNA library preparation. DNA libraries were prepared using the Illumina DNA Prep kit and RNA libraries using the TruSeq Stranded Total RNA v2 kit (Illumina, San Diego, United States), on a TECAN DreamPrep workstation (Tecan Group Ltd., Männedorf, Switzerland). Libraries were paired-end sequenced (2 × 150 bp) on a NovaSeq 6000 system (Illumina, San Diego, United States) with the NovaSeq 6000 S2 Reagent Kit v1.5 (300 cycles). Each metagenomic run included an environmental control (sterile molecular-grade water) and a positive control consisting of a mixture of eight bacterial species and two yeasts (ZymoBIOMICS Microbial Community Standards D6300, Zymo Research, Irvine, United States). Both controls were processed as clinical samples, from pretreatment through bioinformatic analysis.

After sequencing, FASTQ files were analyzed using the in-house patented MetaMIC software v2.3.0. Only samples yielding a cumulative read count (DNA and RNA) of at least 10 million were considered. Adapters were trimmed, as well as low-quality sequences, by removing bases with a Phred score <22 using a 3-nucleotide sliding window. Filtered reads were aligned using Snap-aligner v1.0beta.23 [12] against different databases. First, human sequences were removed after read mapping against the Hg38 genome (NCBI RefSeq assembly GCF_000001405.40). Then, reads matching non-parasitic and non-fungal eukaryotic organisms in the NT database (NCBI, release 06-06-2018) were also removed. The remaining reads were compared with an in-house database based on a filtered and verified version of the NT database (NCBI, release 06-06-2018), and matches were annotated at the species level using the TaxID nomenclature from NCBI. Bacterial identification was considered positive when the corresponding reads exceeded the threshold of 1 sequence per million reads (RPM). Species identification was reported only when detected in both DNA and RNA libraries, as previously described [3]. A sequencing run was considered valid only if all organisms from the positive control were detected. Sequences detected in the environmental control were subtracted from those obtained for each sample.

When reads matched closely related species, DNA and RNA reads were assembled with the option –meta from SPAdes v3.15.5 [13], and the 10 largest contigs were compared against the NCBI NT database (downloaded on April 1, 2023) using BLASTn. A species-level assignment was retained when at least half of these contigs matched the same species; otherwise, only group-level identification was considered.

To further compare the methods in terms of species and group-level assignment, *Streptococcus* species were grouped into *S. mitis* group (*S. mitis, S. oralis, S. pneumoniae, S. pseudopneumoniae*), *S. sanguinis* group (*S. sanguinis, S. gordonii*), β-hemolytic streptococci (*S. agalactiae, S. dysgalactiae, S. pyogenes*), and *S. bovis* group (*S. gallolyticus, S. pasteurianus*) [14].

Lastly, for positive samples, all reads belonging to the same genus as the identified bacteria were mapped against the species reference genome, selected according to the NCBI Genome list (https://www.ncbi.nlm.nih.gov/datasets/genome/) using BWA v0.7.15-r1140 [15]. Coverage and sequencing depth were calculated using SAMtools v1.13-4 [16].

### Statistical Analysis

To compare detection performance between 16S PCR/SS and SMg, a McNemar test was performed on paired samples according to IE classification (definite, possible, or rejected). Assignment performance was compared using a McNemar test over the whole sample collection. The null hypothesis was rejected for *P*-value ≤.05.

## RESULTS

### Included Cases

During the inclusion period, 551 patients with a suspected IE were identified from the IE Observatory database (Figure 1). After applying exclusion criteria, 65 patients were included (37 from Nancy University Hospital and 32 from Henri Mondor University Hospital), of whom 5, 24, and 36 were classified as definite, possible, and rejected IE, respectively, based on major and minor criteria of the 2015 ESC guidelines (Supplementary Table S1). Among them, 72% (47/65) were men and the median age was 66 (IQR = 21). Overall, 69 cardiac samples were available, with four patients having two surgical specimens. Except for two tricuspid vegetations, all other specimens came from the left heart and included aortic valves (24/69, 35%), mitral valves (24/69, 35%), aortic vegetations (13/69, 19%), and mitral vegetations (6/69, 9%).

**Figure 1.**
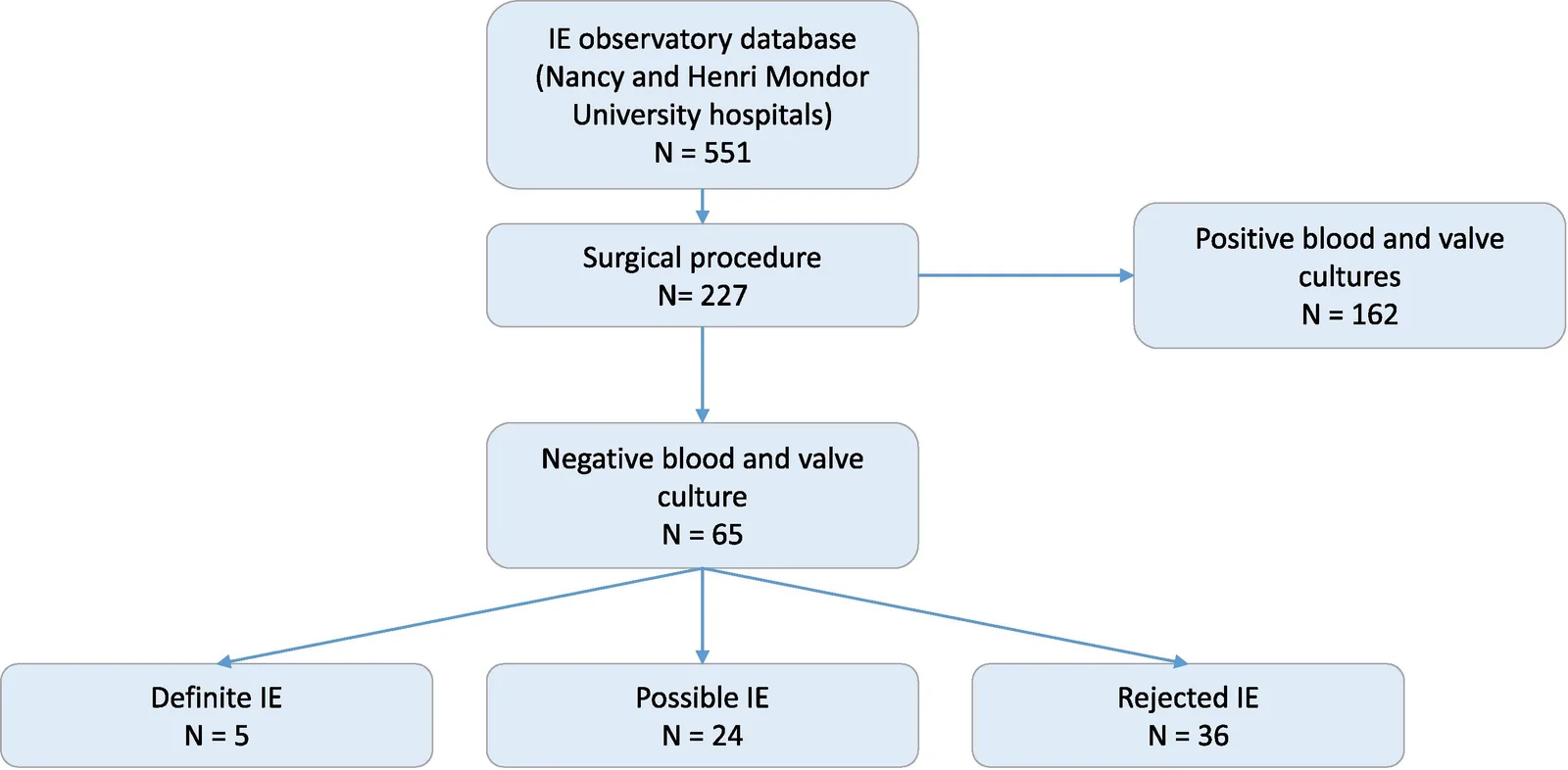
Flowchart of the study. Among the 65 included patients, four had two cardiac samples available, leading to a total of 69 samples analyzed.

### 16S PCR/SS

Out of the 69 samples, 36.2% were positive by 16S PCR/SS (25/69), providing documentation in 36.9% (24/65) of patients: 3 with definite, 15 with possible, and 6 with rejected IE. Among the positive samples, 16S PCR/SS allowed species-level identification in 72% (18/25): *Streptococcus agalactiae* (*n* = 3), *Streptococcus dysgalactiae* (*n* = 3), *Bartonella quintana* (*n* = 3), *Streptococcus gordonii* (*n* = 2), *Streptococcus gallolyticus* (*n* = 2), as well as *Streptococcus pasteurianus*, *Streptococcus pyogenes, Streptococcus sanguinis, Cutibacterium acnes*, and *Proteus mirabilis* each identified once (Figure 2A and 2B, Supplementary Table S2). For the 7/25 (28%) remaining positive samples, identification was obtained at the group level: *Streptococcus mitis/oralis* (*n* = 6) and *Streptococcus pneumoniae/pseudopneumoniae* (*n* = 1), all part of the *S. mitis* group.

**Figure 2.**
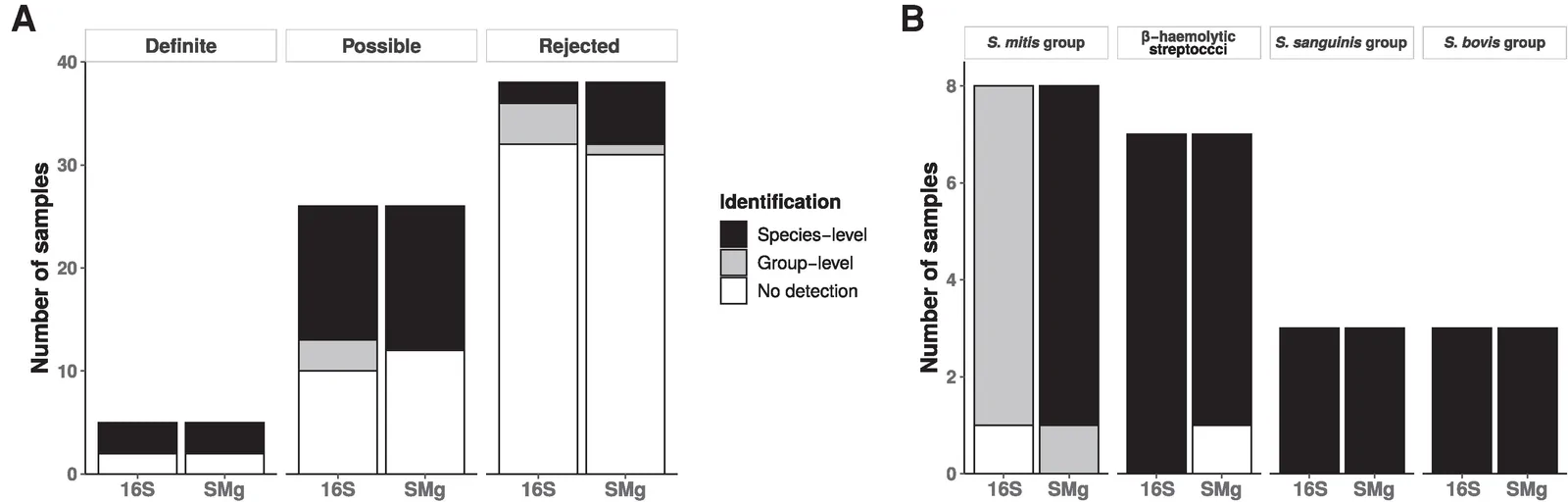
Comparison of 16S PCR/SS and SMg assignment performance according to (A) IE classification and (B) *Streptococcus* taxonomic groups.

### Shotgun Metagenomics

Reads with a minimal Phred quality score of Q30 represented 81.6% of sequences. The median sequencing depths were 34,656,520 DNA reads ([144 470–69 290 052]) and 34,874,810 RNA reads ([14 331 148–61 178 488]). Three samples from three different patients yielded polymicrobial results consistent with contamination, and were therefore uninterpretable and counted as negative. Overall, 34.8% of the samples were positive (24/69), providing documentation in 35.4% (23/65) of patients: 3 with definite, 13 with possible, and 7 with rejected IE. Identification at the species level was achieved for 95.8% (23/24) of positive samples: *Streptococcus oralis* (*n* = 4), *S. dysgalactiae* (*n* = 3), *B. quintana* (*n* = 3), *S. agalactiae* (*n* = 2), *S. gallolyticus* (*n* = 2), *S. mitis* (*n* = 2), *S. gordonii* (*n* = 2), and *S. pasteurianus*, *S. pyogenes*, *S. sanguinis*, *S. pneumoniae*, and *P. mirabilis* each identified once (Figure 2A and B, Supplementary Tables S2 and S3). Only one sample could be identified at the group level only, yielding a *S. mitis/oralis* (*S. mitis* group).

We then calculated the coverage of the RefSeq reference genome of each identified species, as well as the mean sequencing depth (Supplementary Table S4). Among the 24 positive samples, median genome coverage was 11.7% (IQR = 43.9%). Coverage exceeding 90% was obtained in only four samples: two positives for *B. quintana*, one for *S. gordonii*, and one for *S. dysgalactiae*. Mean sequencing depth was also low, with a median of 0.3× (IQR = 3.5×). Only the four samples with high coverage showed a mean sequencing depth exceeding 10×.

### 16S PCR/SS vs SMg

Both methods gave concordant results for 95.7% of the samples (66/69), with 23 concordant positive and 43 concordant negative results (Figure 2A). The three SMg-contaminated samples were negative with 16S PCR/SS. Considering the entire sample collection, no significant difference in positivity rates was observed between the two methods. Likewise, no significant difference was observed at the patient level, regardless of IE classification. 16S PCR/SS only positive samples included one *C. acnes* (possible IE) and one *S. agalactiae* (definite IE). Of note, *S. agalactiae* was detected by SMg, but reached only 0.5 RPM, below the defined threshold. Conversely, the only sample positive by SMg alone yielded a *S. mitis/oralis* (*S. mitis* group) (possible IE). Consistent with this finding, the RefSeq reference genome coverage for this sample was only 1.5%, with a mean sequencing depth of 0.07× (Supplementary Table S4).

Considering samples positive with both methods, SMg performed best at the species level with 23/23 vs 16/23 for the 16S rDNA (*P* = .02). Differences were all observed in the *S. mitis* group (Figure 2B).

## DISCUSSION

In this study, we compared two molecular approaches on surgical samples for the diagnosis of native valve BCNIE, including definite, possible, and rejected IE cases. Only culture-negative surgical samples were analyzed, as this represents the clinical setting in which these methods are most often used. We found similar positivity rates for 16S PCR/SS and SMg for pathogen detection. Interestingly, as both methods were applied to the same nucleic acid extracts, these results likely reflect their intrinsic performance and are consistent with the high diagnostic value of 16S sequencing in IE, whether based on Sanger sequencing [17] or NGS approaches [18].

Only three discrepancies were observed: two samples were positive by 16S PCR/SS and negative by SMg, whereas one showed the opposite pattern. Two were likely related to low bacterial DNA burden. In one case, *S. agalactiae* reads were detected but remained below the reporting threshold. In the other, the group-level assignment obtained by SMg (*S. mitis* group) also suggested a low bacterial DNA burden, potentially explaining the negative 16S PCR/SS result. The third discrepant case yielded *C. acnes*, a skin commensal; therefore, contamination during 16S PCR/SS cannot be completely excluded in this possible IE case involving a native valve.

Of note, SMg results were also interpreted as contamination in two other samples. Although both 16S PCR/SS and SMg are susceptible to contamination, the untargeted nature of SMg and the high sequencing depth may increase the likelihood of detecting environmental contaminants, highlighting the need for careful interpretation and appropriate environmental controls [19]. Moreover, although both approaches were performed from the same DNA extract, 16S PCR/SS and SMg were conducted at two different centers, Nancy University Hospital and Henri Mondor University Hospital, respectively, potentially resulting in differences in exposure to environmental and laboratory contaminants.

In terms of taxonomic resolution, SMg outperformed 16S PCR/SS for species-level assignment, particularly within the *S. mitis* group. This limitation of 16S-based approaches is well recognized [20], but likely has limited clinical consequences, as treatment of oral streptococcal IE relies primarily on penicillin susceptibility rather than species identification [21]. Nevertheless, improved taxonomic resolution may be useful, for example in relapse investigations or cases with only a single positive blood culture.

The existing literature comparing 16S PCR/SS and SMg reports conflicting results, with either higher [6] or lower [7] sensitivity for SMg, while generally showing better taxonomic resolution with SMg. However, sensitivity estimates are difficult to compare with our results because of differences in clinical sample types and technical factors. These studies did not specifically focus on surgical samples from patients with IE, and the two approaches were not compared using the same DNA extract.

Beyond sensitivity and taxonomic resolution, other parameters may also influence the choice between 16S PCR/SS and SMg. For example, SMg may provide advantages in fungal IE, a scenario we were unable to evaluate, likely because only native valves were considered. Its unbiased nature allows the detection of non-bacterial pathogens, although its sensitivity remains lower for fungi than for bacteria [3]. In addition, although polymicrobial IE is rare, Sanger sequencing is not suited for its diagnosis, whereas SMg or NGS-based 16S approaches could better address such situations [1]. Therefore, in cases of negative 16S PCR results in prosthetic valve endocarditis or IE in IV drug users, SMg may provide added value.

Other factors include turnaround time (TAT) and cost. We did not formally evaluate these variables, but the TAT of 16S PCR/SS is often shorter than that of SMg, particularly with automated 16S approaches [22]. At Nancy University Hospital, the TAT of 16S PCR/SS is usually 1 or 2 days, whereas SMg at Henri Mondor University Hospital is typically performed in 6 days [9]. These values may vary considerably depending on local laboratory organization and availability, which may be at least as important as the purely technical processing time. Regarding cost, SMg is more expensive than 16S PCR/SS, with an estimated reagent cost approximately three times higher (around €300 per sample). However, as sequencing costs continue to decrease, the difference between the two approaches may progressively narrow.

Future developments in sequencing and bioinformatics could further enhance the usefulness of SMg, particularly for the detection of resistance and virulence genes [3]. As SMg is an untargeted approach, it theoretically allows the detection of multiple genetic determinants, with potential applications extending beyond taxonomic classification. However, as shown by the very low coverage and sequencing depth observed in our study, such applications would generally require an increase in sequencing depth. While this may be valuable in specific cases, it would probably not be feasible as a first-line strategy for all samples. Moreover, some antibiotic resistance mechanisms, particularly for β-lactams in *Streptococcus* spp., are associated with mutations in core genes rather than the acquisition of resistance genes, which limits our ability to predict the resulting resistance phenotype.

Lastly, among the newly developed SMg approaches, microbial cell-free DNA (mcf-DNA) sequencing directly from blood samples [23, 24] has recently been proposed and incorporated into the 2023 Duke-ISCVID criteria [2], alongside SMg from surgical samples. However, while positive mcf-DNA results for *Coxiella burnetii*, *Bartonella* spp., and *Tropheryma whipplei* are considered major criteria, positivity for other bacterial species carries less diagnostic weight and is classified as a minor criterion. In contrast, positive SMg and 16S-based approaches (PCR/SS or NGS) on cardiac samples are pathologic criteria and are sufficient to establish the diagnosis of IE. Thus, rather than replacing molecular testing on surgical samples, mcf-DNA from blood should probably be considered a complementary approach, with advantages such as its noninvasive nature and longer persistence of positivity than blood cultures, but also limitations including the interpretation of positive results, availability, and cost. Further studies are required to better define the respective indications of each of these approaches.

Our study has several limitations. First, major IE pathogens such as staphylococci and enterococci were absent from our dataset, likely because these bacteria more readily grow from blood culture and valve samples and were therefore excluded. Moreover, the prospective multicenter design provides an unbiased sample of BCNIE cases encountered during the study period. Second, we did not evaluate the impact of antibiotic treatment duration before sampling, which may affect 16S PCR results [25]. Further work is needed to determine whether SMg is similarly affected. Third, we applied a predefined threshold for interpreting SMg results. Such an approach involves a tradeoff between sensitivity and specificity and may result in false negative results, as illustrated by the detection of *S. agalactiae* below this threshold. However, it ensures consistent interpretation across samples.

In conclusion, both 16S PCR/SS and SMg appear equally suitable for the diagnosis of BCNIE, and their use should mainly depend on local availability and expertise. Although less discriminatory, 16S PCR/SS remains a valuable diagnostic tool, except in rarer fungal or polymicrobial IE.

## Supplementary Material

ofag720_Supplementary_Data

## Supplementary Data

Supplementary materials are available at *Open Forum Infectious Diseases* online. Consisting of data provided by the authors to benefit the reader, the posted materials are not copyedited and are the sole responsibility of the authors, so questions or comments should be addressed to the corresponding author.
